# Potentiation of neuritogenic activity of medicinal mushrooms in rat pheochromocytoma cells

**DOI:** 10.1186/1472-6882-13-157

**Published:** 2013-07-04

**Authors:** Syntyche Ling-Sing Seow, Murali Naidu, Pamela David, Kah-Hui Wong, Vikineswary Sabaratnam

**Affiliations:** 1 Mushroom Research Centre, Faculty of Science, University of Malaya, 50603 Kuala Lumpur, Malaysia; 2 Institute of Biological Sciences, Faculty of Science, University of Malaya, 50603 Kuala Lumpur, Malaysia; 3 Department of Anatomy, Faculty of Medicine, University of Malaya, 50603 Kuala Lumpur, Malaysia

**Keywords:** *Ganoderma lucidum*, *Ganoderma neo-japonicum*, *Grifola frondosa*, Neuritogenesis, Neurodegenerative disease, Nerve growth factor, MEK/ERK signaling pathway, PI3K/Akt signaling pathway

## Abstract

**Background:**

Senescence of the neurons is believed to be a focal factor in the development of age-related neurodegenerative diseases such as Alzheimer’s disease. Diminutions in the levels of nerve growth factor (NGF) lead to major declines in brain cell performance. Functional foods, believed to mitigate this deficiency, will be reaching a plateau in the near future market of alternative and preventive medicine. In the search for neuroactive compounds that mimic the NGF activity for the prevention of neurodegenerative diseases, the potential medicinal values of culinary and medicinal mushrooms attract intense interest.

**Methods:**

Cytotoxic effects of aqueous extracts of three medicinal mushrooms basidiocarps, *Ganoderma lucidum*, *Ganoderma neo*-*japonicum* and *Grifola frondosa* towards rat pheochromocytoma (PC-12) cells were determined by 3-(4,5-dimethythiazol-2-yl)-2,5-diphenyltetrazolium bromide (MTT) assay. The potentiation of neuritogenic activity was assessed by neurite outgrowth stimulation assay. Involvement of cellular signaling pathways, mitogen-activated protein kinase kinase/extracellular signal-regulated kinase (MEK/ERK1/2) and phosphoinositide-3-kinase/protein kinase B (PI3K/Akt) in mushrooms-stimulated neuritogenesis were examined by using specific pharmacological inhibitors. Alteration of neuronal morphology by inhibitors was visualized by immunofluorescence staining of the neurofilament.

**Results:**

All the aqueous extracts tested caused a marked stimulation of neuritogenesis with no detectable cytotoxic effects towards PC-12 cells. The aqueous extract of *G*. *neo*-*japonicum* triggered maximal stimulation of neurite outgrowth at a lower concentration (50 μg/ml) with 14.22 ± 0.43% of neurite-bearing cells, compared to *G*. *lucidum* and *G*. *frondosa* that act at a higher concentration (75 μg/ml), with 12.61 ± 0.11% and 12.07 ± 0.46% of neurite-bearing cells, respectively. The activation of MEK/ERK1/2 and PI3K/Akt signaling pathways were necessary for the NGF and aqueous extracts to promote neuritogenesis.

**Conclusions:**

*Ganoderma lucidum*, *G*. *neo*-*japonicum* and *G*. *frondosa* may contain NGF-like bioactive compound(s) for maintaining and regenerating the neuronal communications network. The present study reports the first evidence of the neuritogenic effects of aqueous extracts of basidiocarps of *G*. *neo*-*japonicum in*-*vitro* and showed the involvement of MEK/ERK1/2 and P13K/Akt signaling pathways for neuritogenesis in PC-12 cells.

## Background

According to the World Health Organization (WHO), nearly 35.6 million people worldwide live with dementia in 2010. The number is expected to double by 2030 (65.7 million) and more than triple by 2050 (115.4 million) [1]. Dementia is a brain function syndrome characterized by a cluster of symptoms and signs manifested by difficulties in memory, disturbances in language, psychological and psychiatric changes, and impairments in activities of daily living. Alzheimer's disease is one form of dementia that gradually gets worse over time. It affects memory, thinking, and behaviour [2].

Neuritogenic activity is one of the focuses of the study on the preventive and therapeutic effects of neurodegenerative diseases. Neuritogenic substances hold the promise of therapeutic efficacy in the treatment of neuronal injuries by the virtue of their ability to stimulate outgrowth of neurites from neuronal cells [3]. Recent reports showed that many extracts or compounds from natural sources possessed significant neuritogenic activity *in vitro* and *in vivo*, included hericenones and erinacines from *Hericium erinaceus* (lion’s mane mushroom) [4] and curcumin from *Curcuma longa*[5].

Nerve growth factor has potent biological activities such as promoting neuronal survival and neuritogenesis [6]. It is targeted as a potential therapeutic drug for the treatment of neurodegenerative disorders [7,8]. However, NGF is unstable and is unable to cross blood–brain barrier because of its high molecular polypeptide [9]. Hence, the potential medicinal values of culinary and medicinal mushrooms have attracted intense interest in the search for pharmacological compounds that mimic the NGF activity in the prevention of neurodegenerative diseases. Medicinal mushrooms have a long and rich history of use as mycomedicinals [10,11]. Extracts of medicinal mushrooms have long been an important part of traditional oriental medicines. Many studies reported that edible and medicinal mushrooms possessed neuritogenic effects. In the previous studies, the neuritogenic and nerve regeneration effects of *Hericium erinaceus* (Bull.:Fr.) Pers. in *in vitro* and *in vivo*[12-14], the sclerotium of *Lignosus rhinocerotis* (Cooke) Ryvarden (tiger’s milk mushroom) [15] and *Pleurotus giganteus* (Berk.) Karunarathna & K.D. Hyde (morning glory mushroom) [16] were documented.

The genus *Ganoderma* is a popular medicinal mushroom, and is used in traditional Chinese medicine (TCM) as a tonic and sedative in Asian countries. For over two millennia its use is documented in countries including China, Japan and Korea [17,18]. *Ganoderma lucidum* (Curtis: Fr.) P. Karst, called “Lingzhi” in Chinese and “Reishi” in Japanese, is one of the most commonly used mushroom by TCM in Asia [17]. According to “Shennong Ben Cao Jing”, a Chinese book on agriculture and medicinal plants (300 BC – 200 AC), Lingzhi is classified into six categories based on colour, which are red, yellow, black, white, green and purple. *Ganoderma lucidum* is the most common red Lingzhi and *Ganoderma neo*-*japonicum* Imazeki is categorized as purple Lingzhi. *Ganoderma neo*-*japonicum* is found in Mainland China, Japan and Taiwan, and grows saprotrophically on dead hardwoods or bamboos [19]. In Malaysia, *G*. *neo*-*japonicum* grows on bamboo. A water infusion is used by the indigenous folks as medicine and a tonic to strengthen the body (unpublished data). *Grifola frondosa* (Dicks.) Gray, also known by its Japanese name “Maitake” which means “dancing mushroom”, has been used as a health food for centuries in China and Japan. Maitake is a delicious culinary mushroom and also valued for its medicinal properties. Studies have shown that *G*. *lucidum*[18] and *G*. *frondosa*[20] possessed neuritogenic effects in preventing and treating neurological disorders. However, no information is available on the neuronal effects of *G*. *neo*-*japonicum*.

The present work reports the study of neuritogenic effects of aqueous extracts of medicinal mushrooms basidiocarps, namely *H*. *erinaceus*, *G*. *lucidum*, *G*. *neo*-*japonicum* and *G*. *frondosa* on PC-12 cells. Furthermore, the effects of cellular signaling pathways, MEK/ERK1/2 and PI3K/Akt in the potentiation of neuritogenic activity in PC-12 cells by using specific pharmacological inhibitors were investigated.

## Methods

### Materials and chemicals

The *H*. *erinaceus* (KLU-M 1232) and *G*. *lucidum* (KLU-M 1233) basidiocarps were obtained from Ganofarm in Tanjung Sepat, Selangor. *Ganoderma neo*-*japonicum* (KLU-M 1231) basidiocarps were collected from a forest in Ulu Grik, Perak and *G*. *frondosa* basidiocarps (KLU-M 1229) were purchased from a hypermarket in Selangor, Malaysia. The mushrooms were identified and authenticated by experts in the Mushroom Research Centre, University of Malaya. Voucher specimens are deposited in the University of Malaya herbarium (KLU-M). Rat pheochromocytoma (PC-12Adh) cell line was purchased from American Type Culture Collection (ATCC; Rockville, MD, USA; Catalogue Number: CRL-1721.1). Kaighn’s Modification of Ham’s F-12 Medium (F-12 K medium), NGF-7S from murine submaxillary gland, 3-(4,5-dimethythiazol-2-yl)-2,5-diphenyltetrazolium bromide (MTT), phosphate buffered saline (PBS), dimethyl sulfoxide (DMSO), MEK inhibitor (U0126, PD98059), PI3K inhibitor (LY294002), anti-neurofilament 200 (NF-200) antibody produced in rabbit and Anti-Rabbit IgG-Fluorescein isothiocyanate (FITC) antibody produced in sheep were obtained from Sigma Co. (St. Louis, MO, USA). ProLong® Gold Antifade Reagent with DAPI (4-6-Diamidino-2-phenylindole) was purchased from Life Technologies Corporation (California, USA). Fetal bovine serum (FBS) and horse serum (HS) were purchased from PAA Laboratories (Cölbe, Germany).

### Preparation of aqueous extracts

The aqueous extracts were prepared according to Eik *et al*. [15]. Briefly, the fresh basidiocarps of *H*. *erinaceus* and *G*. *frondosa* were sliced, weighed and freeze-dried while *G*. *lucidum* and *G*. *neo*-*japonicum* were air dried. The dried basidiocarps were then ground into powder by a Waring commercial blender. The powder was then soaked in distilled water at a ratio of 1:20 (w/v) and 150 rpm at room temperature. After 24 h, the mixture was double boiled in a water bath for 30 min and after cooling was filtered through Whatman no. 4 filter paper. The resulting aqueous extracts were freeze-dried and kept at −20°C prior to use.

### *In vitro* cell culture

The rat pheochromocytoma (PC-12Adh) cells were sustained in ATCC formulated F-12 K medium and supplemented with 15% (v/v) of heat-inactivated HS and 2.5% (v/v) of heat-inactivated FBS with final pH 6.8 - 7.2. The cells were subcultured every 2 to 3 days and incubated at 37 ± 2°C in a 5% CO_2_-humidified incubator.

### Cell viability and cytotoxicity assay

Cell viability was assessed by the mitochondrial-dependent reduction of MTT to purple formazan. PC-12 cells were plated in 96-well plates at a density of 5 × 10^3^ cells/well and incubated overnight at 37°C in a 5% CO_2_-humidified incubator. Then, the aqueous extracts (0–2500 μg/ml) were added into the cells. After 48 h of incubation, 20 μl of MTT (5 mg/ml) in PBS buffer (pH 7.4) was added into each well and incubated at 37°C for 4 h. Subsequently, the supernatant was carefully discarded by aspiration, and 100 μl of DMSO was then added into each well to dissolve the MTT formazan crystals, mixed thoroughly and incubated for 15 min. The extent of the reduction of MTT was determined by measurement of the absorbance at 540 nm with 690 nm as background absorbance with an ELISA microplate reader (Sunrise, Tecan, Austria). The complete F-12 K medium was the blank, and cells incubated in the medium only were denoted as the negative control.

### Neurite outgrowth stimulation assay

Cells were plated in 12-well plates at a density of 5 × 10^3^ cells per well in complete F-12 K medium. The cells were treated with freshly prepared aqueous extracts at various concentrations ranged from 25 to 100 μg/ml (w/v). Eik *et al*. [15] reported that 50 ng/ml (w/v) of NGF-7S from murine submaxillary gland was the optimum concentration for neuritogenesis in PC-12 cells. In the present study, cells treated with 50 ng/ml (w/v) of NGF or 50 μg/ml (w/v) of *H*. *erinaceus* served as positive controls. Cells in complete F-12 K medium without treatment served as a negative control. Assay plates were incubated for 48 h at 37 ± 2°C in a 5% CO_2_-humidified incubator.

### Quantification of neurite outgrowth

The cell morphology was assessed under an inverted microscope (Nikon Eclipse TS100). Neurite extension of PC-12 cells was regarded as an index of neuritogenesis. Neurite that was double or more the length of the cell body diameter was scored positive for a neurite-bearing cell [14]. The images were captured with a QImaging Go-3 color CMOS Camera (QImaging, Canada) and by the image processor system, Image-Pro Insight (MediaCybernetics, MD). The percentage of differentiated cells was evaluated by scoring the proportion of neurite-positive cells to total cells in randomly 10 selected microscopic fields per well, with an average of 200–300 cells per well.

### Treatment with specific inhibitors of signaling pathways

The MEK/ERK1/2 inhibitors (U0126, PD98059) and PI3K/Akt inhibitor (LY294002) were used in this study. Stock solutions (10 mM) of inhibitors were prepared in DMSO and stored at −20°C in the dark. Final concentrations of 10 μM of U0126, 30 μM of LY294002 and 40 μM of PD98059 were prepared by diluting in complete F-12 K medium just before use [16]. Cells were pre-incubated either with or without the inhibitor for 1 h at 37 ± 2°C in a 5% CO_2_-humidified incubator, respectively before the treatment with 50 ng/ml (w/v) of NGF or the optimum concentration of each aqueous extract resulting in the neurite outgrowth stimulation assay. Cells were then incubated for 48 h prior to scoring the neurite-bearing cells.

### Immunofluorescence staining of neurofilament

Immunofluorescence assay was carried out according to Schimmelpfeng *et al*. [21] with some modifications. Briefly, cells were seeded in 12-well micro-chamber (ibidi, Martinsried, Germany) at a density of 5 × 10^3^ cells per well in complete F-12 K medium. Then, the cells were pre-incubated either with or without the treatment of inhibitors. After 1 h, the cells were treated with the optimum concentration of each aqueous extract result in the neurite outgrowth stimulation assay for 48 h at 37 ± 2°C in a 5% CO_2_-humidified incubator. Subsequently, the cells were fixed with 4% formalin (v/v) at room temperature for 20 min. After three washings with PBS, the cells were incubated with anti-NF-200 antibody produced in rabbit (1:80 dilution in blocking buffer) at room temperature for 1 h. Then, the cells were incubated with fluorophore-conjugated secondary antibody, anti-Rabbit IgG-FITC antibody produced in sheep (1:80 dilution in blocking buffer) at room temperature for 1 h in the dark. Cells were mounted with aqueous mounting medium, ProLong® Gold Antifade Reagent with DAPI. Slides were observed under fluorescence illumination using FITC and DAPI filters and images were captured with Nikon’s Imaging Software, NIS-Elements.

### Statistical analysis

All the experimental data were expressed as the mean ± standard deviation (SD). Statistical differences between groups were performed using one-way analysis of variance (ANOVA) of a minimum of three independent experiments and Duncan's multiple range tests (DMRT) P < 0.05 was considered to be significant.

## Results

### The cells viability and cytotoxic effects of aqueous extracts on PC-12 cells

**Figure 1:**
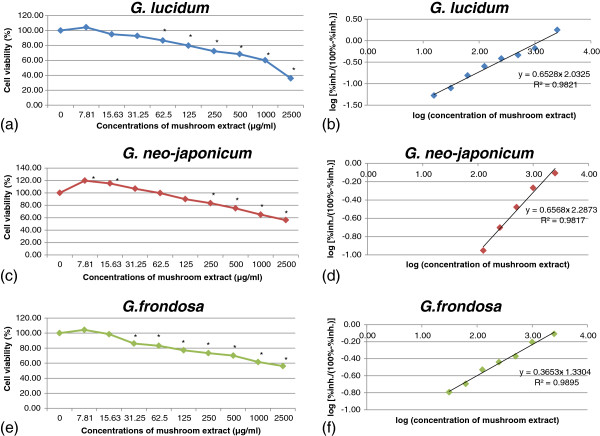
**Effects of aqueous extracts on the cell viability of PC-12 cells.** PC-12 cells were treated with aqueous extracts at concentrations from 0 to 2500 μg/ml for 48 h. **(a)***Ganoderma lucidum*, **(c)***Ganoderma neo*-*japonicum* and **(e)***Grifola frondosa*. **(b)**, **(d)** and **(f)**: IC_50_ was obtained from the intercept on the x-axis (y = 0) of the regression line using the linear part of the percentage inhibition (% inh.) curve. The mean absorbance was obtained using complete F-12 K medium with cells only was designated 100%. Results are shown as means ± standard deviation (n = 3). * p < 0.05 compared to the respective control 100%.

All aqueous extracts tested did not exert any detectable cytotoxic effect in PC-12 cells. The survival rates of the cells were decreased in a concentration-dependent manner, *G*. *lucidum* (Figure 1a,b), *G*. *neo*-*japonicum* (Figure 1c,d), and *G*. *frondosa* (Figure 1e,f). The negative control, cells in complete F-12 K medium only, was considered as 100% of cell viability. A significant (p < 0.05) stimulation of proliferation was observed at the concentration of 7.81 μg/ml and 15.63 μg/ml of *G*. *neo*-*japonicum*. The cell viability was significantly (p < 0.05) decreased at the concentration of 62.5 ug/ml (*G*. *lucidum*), 250 ug/ml (*G*. *neo*-*japonicum*) and 31.25 ug/ml (*G*. *frondosa*) with the percentage inhibitions of 13.41%, 16.57% and 13.85%, respectively, compared to the negative control. The reduction in the cell number could be a consequence of cell death or the decrease in the cell division. The required concentration to inhibit the cell growth by 50% (IC_50_) for aqueous extracts of *G*. *lucidum*, *G*. *neo*-*japonicum* and *G*. *frondosa* were 1298.71 ug/ml, 3037.32 ug/ml and 4384.68 ug/ml, respectively.

### The neuritogenic effect of aqueous extracts on PC-12 cells

**Figure 2:**
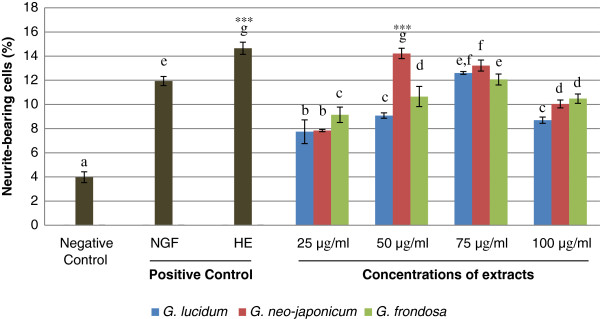
**Effects of aqueous extracts on the neuritogenic activity of PC-12 cells.** The percentage of neurite-bearing cells of PC-12 cells treated with various concentrations of aqueous extracts ranged from 25 to 100 μg/ml. Cells in complete F-12 K medium without treatment served as a negative control. Cells treated with 50 ng/ml of NGF or 50 μg/ml of *H*. *erinaceus* (HE) served as positive controls. Data were expressed as means ± standard deviation (n = 3). Means with different alphabets shows significant difference p < 0.05. *** p < 0.001 compared to NGF.

All concentrations of aqueous extracts tested showed neuritogenic effects after 48 h of incubation (Figure 2). Nerve growth factor- and *H*. *erinaceus*- treated cells served as positive controls. The percentage of neurite-bearing cells of *G*. *lucidum*-, *G*. *neo*-*japonicum*- and *G*. *frondosa*- treated cells were significantly (p < 0.05) increased in a concentration-dependent manner. There were significant differences (p < 0.05) between the negative control and all concentrations of aqueous extracts tested. Interestingly, the percentage of neurite-bearing cells of aqueous extract of *G*. *neo*-*japonicum* at 50 μg/ml (14.22 ± 0.43%) was significantly higher (p < 0.05) compared to NGF and was comparable to neurite outgrowth stimulation by *H*. *erinaceus* (14.66 ± 0.5%). Maximum stimulation of neuritogenesis by aqueous extract of *G*. *neo*-*japonicum* was achieved at 50 μg/ml with 14.22% of neurite-bearing cells, followed by *G*. *lucidum* (12.61%) and *G*. *frondosa* (12.07%) at a higher concentration of 75 μg/ml. There was no significant difference in the percentage of neurite-bearing cells between 50 ng/ml of NGF (11.94 ± 0.38%) and 75 μg/ml of aqueous extract of *G*. *lucidum* (12.61 ± 0.11%) and *G*. *frondosa* (12.07 ± 0.46%).

### The involvement of MEK/ERK1/2 and PI3K/Akt signaling pathways in aqueous extracts-stimulated neuritogenesis

**Figure 3:**
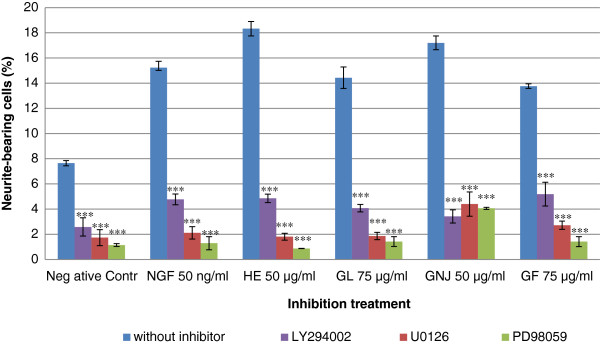
**Effects of the specific inhibitors of MEK/ERK1/2 and PI3K on aqueous extracts-stimulated neuritogenesis.** PC-12 cells were exposed to U0126, PD98059 and LY294002 for 1 h before the treatment of aqueous extracts of *G*. *lucidum* (GL), *G*. *neo*-*japonicum* (GNJ) and *G*. *frondosa* (GF). Cells in complete F-12 K medium without treatment served as a negative control. Cells treated with 50 ng/ml of NGF or 50 μg/ml of *H*. *erinaceus* (HE) served as positive controls. A control without inhibitor was used in each treatment group. Data were expressed as means ± standard deviation (n = 3). *** p < 0.001 compared to respective controls.

The MEK/ERK1/2 inhibitors, U0126 (10 μM) and PD98059 (40 μM) blocked the neuritogenic activity of aqueous extracts and NGF (Figure 3). The results showed that PD98059 decreased the percentage of neurite-bearing cells by approximately 90.16% in *G*. *lucidum*, 76.42% in *G*. *neo*-*japonicum* and 89.73% in *G*. *frondosa* treated cells compared to each individual control. In the presence of PI3K/Akt inhibitor, LY294002 (30 μM), the number of neurite-bearing cells were decreased significantly (p < 0.001). The significant (p < 0.001) reduction of neurite stimulation activities were also observed in the negative control-, NGF- and aqueous extracts of *H*. *erinaceus*- stimulated neuritogenesis with the addition of the inhibitors. These data suggest that activation of MEK/ERK1/2 and PI3K/Akt signaling pathways are involved in aqueous extracts-stimulated neuritogenesis in PC-12 cells.

### The effect of MEK/ERK1/2 and PI3K/Akt inhibitors on neuronal morphology visualized by immunofluorescence staining

**Figure 4:**
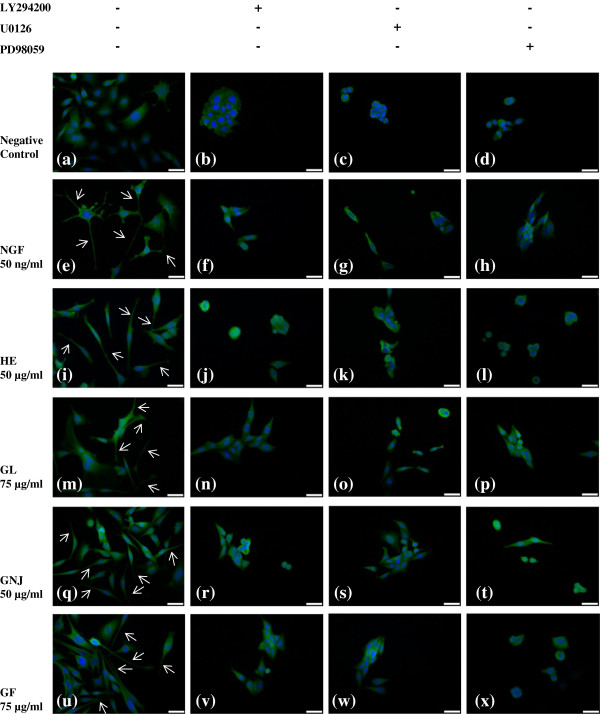
**Morphology of PC-12 cells in different treatment groups.** PC-12 cells were exposed to U0126 (10 μM), PD98059 (40 μM) and LY294002 (30 μM) for 1 h before the treatment of aqueous extracts. **(a)** to **(d)** negative control (complete F-12 K medium), **(e)** to **(h)** positive control (NGF; 50 ng/ml), **(i)** to **(l)** positive control (*H*. *erinaceus* (HE); 50 μg/ml), **(m)** to **(p)** 75 μg/ml of *G*. *lucidum* (GL), **(q)** to **(t)** 50 μg/ml of *G*. *neo*-*japonicum* (GNJ), **(u)** to **(x)** 75 μg/ml *of G*. *frondosa* (GF). Nuclei stained blue and neurofilaments stained green. Scale bars represent 50 μM. Arrows indicate neurite outgrowth.

To examine the pattern of neuritogenesis further, PC-12 cells were stained by immunofluorescence dyes incorporated with anti-NF-200 antibody. PC-12 cells nuclei were stained blue by DAPI and neurofilaments were stained green by anti-NF-200 labeled with FITC. The cells were pre-treated, with or without specific inhibitors, prior to the addition of the aqueous extracts and incubated for 48 h. In the negative control, the cells are relatively small and rounded with few visible neurites (Figure 4a). With the treatment of 50 ng/ml of NGF, 50 μg/ml of *H*. *erinaceus*, 75 μg/ml of *G*. *lucidum*, 50 μg/ml of *G*. *neo*-*japonicum* and 75 μg/ml of *G*. *frondosa*, the cells were larger and elongated. Cells also exhibited neurite extensions that were double the length of the cell body diameter (Figure 4e, 4i, 4m, 4q, 4u). However, some morphological changes in neuronal differentiation were observed in the treatment of U0126, PD98059 and LY294002 inhibitors. The inhibitors blocked the neuritogenic activity of aqueous extracts and NGF and caused shrunken and rounded cell bodies without noticeable neurite extension. These results suggest that the activation of MEK/ERK1/2 and PI3K/Akt signaling pathways are needed for the NGF and aqueous extracts in promoting neuritogenesis.

## Discussion

In the present study, PC-12Adh cell line was utilized as a model system to investigate the cytotoxicity, neuritogenic activity and elucidate the underlying mechanisms of aqueous extracts of medicinal mushrooms basidiocarps, namely *G*. *lucidum*, *G*. *neo*-*japonicum* and *G*. *frondosa*. The PC-12 cell line is established from rat adrenal pheochromocytoma (adrenal medullary tumour) [22] and has been extensively used as a model to investigate the neuronal differentiation, proliferation and survival [23]. With the addition of NGF, PC-12 cells are able to differentiate into sympathetic neuron-like phenotypes characterized by neurite outgrowth and the expression of several neuron-specific proteins [22,24]. Nerve growth factor is crucial for the survival, developmental and differentiation of the central and peripheral neurons [25,26]. The neurotrophic effect of NGF is transduced by high affinity tyrosine receptor TrkA [27], the NGF receptor, and then it activates several signaling pathways via intracellular signaling molecules that include Ras [28], PI3K [26,29], ERK [30] and p38 MAPK [31].

Aqueous extraction has been the most commonly used method for the isolation of bioactive polysaccharides from mushrooms such as glucans [10,32]. According to Cheung *et al*. [18], the extract of *Ganoderma* contained polysaccharides that possessed neuroactivity. It had been reported that crude aqueous extract of *Tremella fuciformis* (white jelly mushroom) possessed neuritogenic effects *in vitro* and anti-amnesic effects *in vivo*[33]. According to Lin *et al*. [34], treatment with the water extract of *G*. *lucidum* and *G*. *neo*-*japonicum* showed antioxidant effect on free radical scavenging activity and hepatoprotective effect against CCl4-induced liver injury. Aqueous extraction is believed to have lower cytotoxic effect compared to most of the organic solvent. In this study, medicinal mushrooms were extracted by water, in conjunction with the traditional use of mushrooms as part of TCM. In addition, water is non-toxic to cells. From data obtained in this study, the IC_50_ value of cytotoxic activity of *G*. *lucidum*, *G*. *neo*-*japonicum* and *G*. *frondosa* were approximately 17-, 60- and 58- fold higher than their optimum concentration that stimulated neuritogenesis. Further, the results indicated that the aqueous extracts of all tested mushrooms were not cytotoxic to PC-12 cells.

The results suggested that all aqueous extracts tested caused a marked stimulation of neuritogenesis in PC-12 cells and they appeared to be comparably active with the neuritogenic effects *in vitro* of NGF. Therefore, the aqueous extracts of *G*. *lucidum*, *G*. *neo*-*japonicum* and *G*. *frondosa* may possess NGF-like bioactive compounds that mimic the neuroactivity of NGF for neuronal survival, development and differentiation. The aqueous extract of *G*. *neo*-*japonicum* triggered maximal stimulation of neuritogenesis at a lower concentration compared to *G*. *lucidum* and *G*. *frondosa* that act at a higher concentration. Neuritogenic activity of higher basidiomycetes other than *G*. *neo*-*japonicum*, *G*. *lucidum* and *G*. *frondosa* has also been reported. These included *H*. *erinaceus*[4,35], *Sarcodon scabrosus*[36], *Sarcodon cyrneus*[37,38], *Termitomyces albuminosus*[39,40] and *Cordyceps militaris*[41]. Shi *et al*. [36] reported that cyathane diterpenoids isolated from *S*. *scabrosus* showed significant neuritogenic activity in combination with 20 ng/mL of NGF in PC-12 cells after 24 h treatment. The extract of *C*. *militaris* stimulated neuritogenesis, enhanced neuronal functions of Neuro2A mouse neuroblastoma cells (*in vitro*) and improved cognitive behaviour that related to memory ability (*in vivo*) [41].

Our findings illustrated the potential cellular signaling pathways involved in aqueous extracts-stimulated neuritogenesis, namely MEK/ERK1/2 and P13K/Akt that are important in regulating growth and differentiation of PC-12 cells. Specific inhibitors of MEK/ERK1/2 and P13K/Akt could attenuate the ability of aqueous extracts to stimulate neuritogenesis in PC-12 cells. The MEK/ERK and PI3K/Akt signaling pathways can be activated by NGF to stimulate neurite extension and branching of neuronal cells [42-45]. Vaudry *et al*. [30] reported that the activation of MEK/ERK signaling pathway is necessary for neuritogenesis, in this case the neuronal differentiation in PC-12 cells by NGF. Inhibition of PI3K in PC-12 cells will avert NGF-stimulated neurite elongation [25], promote cell protective effect and cell survival [46].

**Figure 5:**
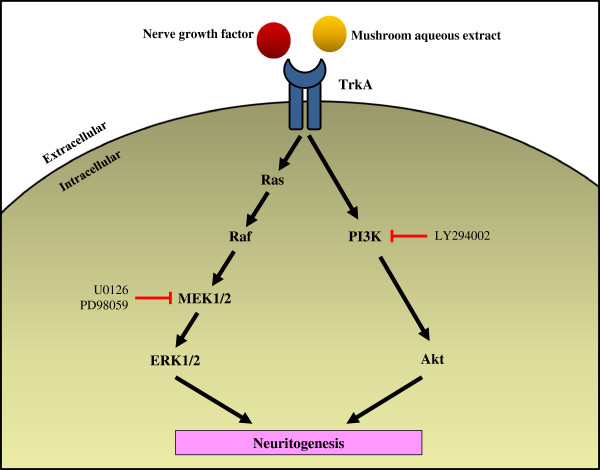
**Hypothetic mechanisms of NGF and mushroom aqueous extracts in promoting neuritogenesis in PC-12 cells.** Both nerve growth factor and aqueous extracts bind to tyrosine receptor, TrkA and initiate two major signaling pathways, the MEK/ERK1/2 and PI3K/Akt pathways. Activation of MEK/ERK1/2 and PI3K/Akt cascade promotes neuritogenesis of PC-12 cells. In the present study, the MEK/ERK1/2 inhibitors (U0126, PD98059) and PI3K/Akt inhibitor (LY294002) blocked the neuritogenic activity of NGF and aqueous extracts. Therefore, based on the present findings, the activation of MEK/ERK1/2 and PI3K/Akt signaling pathways are needed for the NGF and aqueous extracts in promoting neuritogenesis.

In this study, the potentiation of aqueous extracts-stimulated neuritogenesis was blocked by U0126, PD98059 and LY294002. Therefore, the MEK/ERK and PI3K/Akt-dependent signaling pathways play a crucial role in the neuritogenic effect of medicinal mushrooms (Figure 5). This is in agreement with a previous study by Phan *et al*. [16], documented that MEK/ERK and PI3K/Akt signaling pathways were involved in neuritogenesis stimulated by extracts of *P*. *giganteus*. Some studies have shown the involvement of MAPK cascade in neuritogenesis. Extracts of *Ganoderma*[18] and lysophosphatidylethanolamine, a neuroactive compound isolated from *G*. *frondosa*[20] activated the MAPK cascade through the MEK/ERK1/2 phosphorylation of PC-12 cells.

Neurofilament staining by immunofluorescence served as firm support to the observation that aqueous extracts-stimulated neuritogenesis. Neurofilament is a neuron-specific protein that serves as a major component of the cytoskeleton that supporting the axon cytoplasm. It is a useful indicator of PC-12 cell differentiation [21]. The images showed clear morphological differences between the inhibitor-treated and non-inhibitor-treated groups. The addition of the MEK/ERK or PI3K/Akt inhibitors blocked the neuritogenesis of PC-12 cells and the neurite outgrowth of the NGF- and aqueous extracts-stimulated PC-12 cells.

Besides MEK/ERK1/2 and PI3K/Akt, other mechanisms may still be addressed for a comprehensive understanding of neuritogenic activity. The interaction between MEK/ERK and PI3K/Akt signaling pathways determined by flow cytometry or immunoblot analysis will be proposed for elucidation of mechanisms involved in the neuritogenic activity of the three selected mushrooms.

## Conclusions

Our findings suggested that all the medicinal mushrooms tested possessed neuritogenic activity without cytotoxic effect. The MEK/ERK1/2 and PI3K/Akt signaling pathways may play a role in the neuritogenic activity of the mushrooms. The precise mechanism underlying this activity remains to be investigated.

## Abbreviations

Akt: Protein kinase B; ERK: Extracellular signal-regulated kinase; MAPK: Mitogen activated protein kinase; MEK: Mitogen-activated protein kinase kinase; P13K: Phosphoinositide-3-kinase.

## Competing interests

The authors declare that they have no competing interests.

## Authors’ contributions

SLSS carried out the experiment, drafted the manuscript, engaged in data acquisition and data interpretation. MN participated in the acquisition of funding and editing of the manuscript. PD and KHW were involved in the design of the study and manuscript editing. VS provided the grant, was involved in coordinating and monitoring of research, and manuscript editing. All authors read and approved the final manuscript.

## Pre-publication history

The pre-publication history for this paper can be accessed here:

http://www.biomedcentral.com/1472-6882/13/157/prepub
